# Role of aspirin, beta-blocker, statins, and heparin therapy in septic patients under mechanical ventilation: a narrative review

**DOI:** 10.3389/fmed.2023.1143090

**Published:** 2023-07-10

**Authors:** Lou'i Al-Husinat, Amer Abu Hmaid, Hadeel Abbas, Batool Abuelsamen, Mutaz Albelbisi, Said Haddad, Ibrahim Qamileh, Ossaid Quneis, Zaid Jehad Al Modanat, Giuseppe Ferrara, Fernando Suparregui Dias, Gilda Cinnella

**Affiliations:** ^1^Department of Clinical Medical Sciences, Faculty of Medicine, Yarmouk University, Irbid, Jordan; ^2^Faculty of Medicine, Yarmouk University, Irbid, Jordan; ^3^Faculty of Medicine, Jordan University of Science and Technology, Irbid, Jordan; ^4^Department of Anesthesia and Intensive Care, Al-Maqasid Charity Hospital, Amman, Jordan; ^5^Department of Anesthesia and Intensive Care, University of Foggia, Foggia, Italy; ^6^Department of Intensive Care, Hospital São Lucas da PUCRS, Porto Alegre, Brazil

**Keywords:** sepsis, aspirin, beta-blocker, statin, heparin

## Abstract

Sepsis is the main cause of death among patients admitted to intensive care units. Management of sepsis includes fluid resuscitation, vasopressors, intravenous antimicrobials, source control, mechanical ventilation, and others. New insights into the potential benefits of non-antimicrobial drugs in sepsis have evolved based on the pathophysiology of the disease and the mechanism of action of some drugs, but the findings are still controversial. In this study, we aimed to evaluate the effect of beta-blockers, aspirin, statins, and heparin as adjunctive treatments in septic patients under mechanical ventilation with non-cardiovascular diseases and their effect on mortality. We searched PubMed with relevant keywords (beta-blockers, aspirin, statins, or heparin, and critically ill or sepsis) for the last 10 years and some personal collection of relevant articles, and then we assessed studies according to prespecified inclusion and exclusion criteria. Our results show that beta-blockers, aspirin, and heparin may have promising feedback on reducing mortality. However, new well-controlled, randomized, multicenter studies are needed to confirm that, and multiple issues regarding their usage need to be addressed. On the other hand, the feedback regarding the effectiveness of statins was not as strong as that of the other drugs studied, and we suggest that further research is needed to confirm these results.

## Introduction

Modern sepsis definition is characterized as a severe organ dysfunction caused by an impaired host immune response to infection. Clinically, sepsis is defined by a documented focus of infection with at least 2 points or more in the Sequential Organ Failure Assessment (SOFA) (1) score. On the other hand, septic shock is an advanced sepsis stage with circulatory and metabolic derangements enough to increase mortality (2). Sepsis represented 19.7% of all global deaths with approximately 49 million incident cases in 2017 (3). In developing countries, sepsis incidence *is* estimated to be from 22 to 240/100 000 with a mortality rate of up to 30 %, which depends on the setting and disease severity (4). The cornerstone of sepsis management is supportive measures along with the administration of antimicrobial agents, source control, in addition to hemodynamic support when shock is present (5). Despite advances in care and recognition, sepsis remains a global public health concern due to its high mortality, high health-related risk, and economic burden (6).

As sepsis causes intense immunological, inflammatory, and coagulation derangements, in the last few years, therapies that could ameliorate organ dysfunction/failure in this setting were tested in these patient groups. Studies aimed at modulating and restoring the coagulation, immunological, and inflammatory response were conducted utilizing specific molecules, such as recombinant tissue factor pathway (7), recombinant C-protein (8), or synthetic endotoxin-like immunomodulators (9) but the results were disappointing.

Facing this scenario, successful therapies directed at other diseases were examined in septic patients, such as beta-blockers, aspirin, heparin, and statins, considering their biological plausibility in this setting.

As inflammation can augment myocardial oxygen consumption by excessive adrenergic stimulation, the role of beta-blockade has been studied focusing abate cardiovascular stress. However, there is a scarcity of data to confirm that these drugs could improve survival (10).

Platelets have an important role in sepsis pathophysiology, leading researchers to examine the role of drugs that act in the coagulation system, such as aspirin and heparin (11, 12).

In an *in vivo* study, pretreatment with simvastatin blunted the effects of TLR4 and TLR2 after a challenge with intravenous LPS in 20 healthy male subjects. These results suggested that statins may have a suppressive effect on these receptors and reduce cytokine actions (13) and that 80 mg of simvastatin for 7 days is associated with better outcomes in patients with community-acquired pneumonia (CAP) (14). Considering these possible beneficial effects, statins are studied in critically ill or septic patients to test if these results could be reproduced in the clinical scenario. The results were mixed (15, 16), and two recent meta-analyses showed no beneficial effect of this drug category in sepsis (17, 18).

Despite these findings, clinical research on beta-blocker, aspirin, heparin, and statins therapies in septic patients is still controversial, as the outcomes remain unclear and require further study. This study aimed to summarize the evidence on the use of these drugs on the outcome and prognosis in septic adult patients.

## Methods

### Data source and searches

Literature searches were performed using PubMed on 8 August 2022 and included the literature since 2012 as well as a personal collection of relevant articles.

### Study selection

Titles were scanned and abstracts were reviewed in duplicate (AAH and HA). In the second stage, full-texts were examined. Any disagreements were adjudicated using a third reviewer (FSD).

Studies that compared heparin, aspirin, statins, and or beta-blockers to standard treatment in septic ICU patients were included. Search terms included: beta-blockers OR aspirin OR statins OR heparin AND critically ill OR sepsis; 141 primary references were obtained. The inclusion criteria were as follows:

(1) study population consisted of septic patients with non-cardiovascular diseases treated in the ICU, (2) studies published in the time frame between 2012 and 2022, (3) outcome of illness was measured, and (4) the language of studies in English only. The exclusion criteria were as follows: pregnant women and those less than 18 years of age were excluded.

Retrospective investigations, prospective observational studies, meta-analysis, systematic review, and randomized controlled trials were included; literature reviews were excluded.

It is worth mentioning that our study design, narrative review, does not follow a specific protocol, and no recommendations for clinical practice are needed.

### Data extraction and quality assessment

The data were extracted and tabulated in duplicate (AAH and HA) using data collection headings as follows: article title, first author, place and year, study type/design, outcome, and intervention.

The certainty of evidence for each outcome was assessed using the Grading of Recommendations, Assessment, Development, and Evaluation (GRADE) framework (19).

## Results

**Figure 1:**
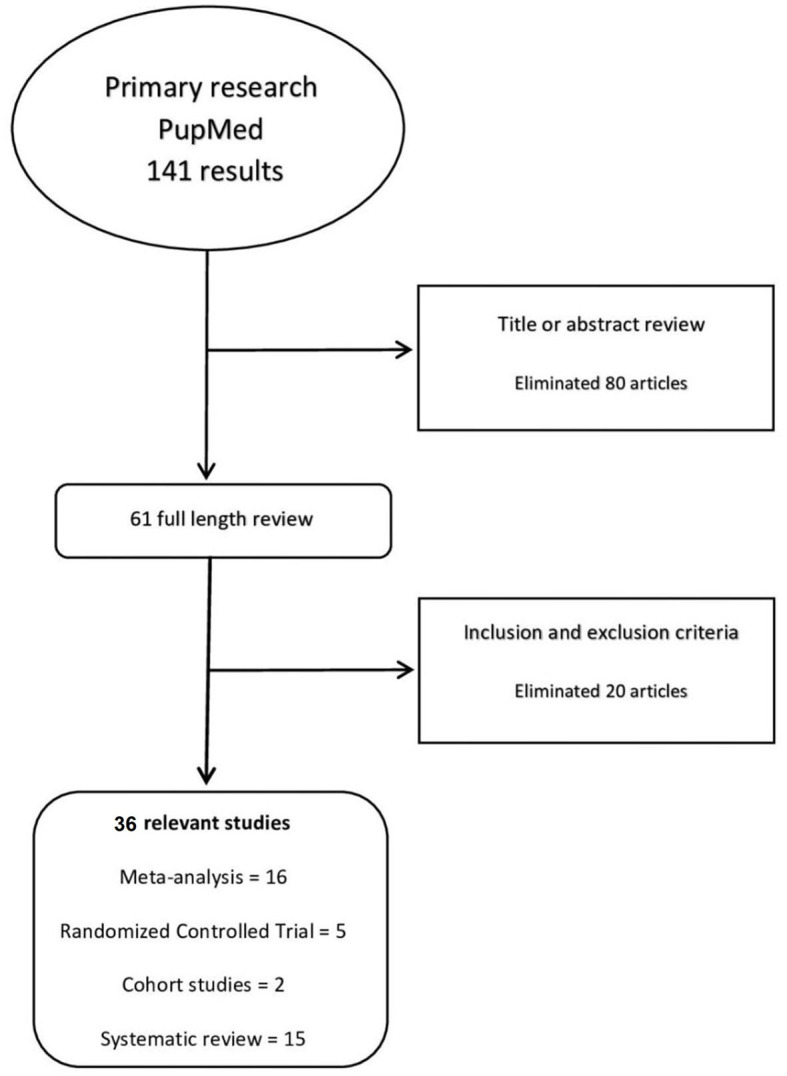
Methods: process of review.

We reviewed 141 citations, reviewed 61 full-texts, and included 36 studies (*n* = 1,969,965) which showed in Figure 1.

The characteristics of most of the included studies are reported in Tables 1–3.

**Table 1:** Included systematic reviews and meta-analysis studies regarding beta-blockers.

**References**	**Study design**	**Number of participant s [n]**	**Results**	**Outcomes**
Heliste et al. (20)	Systematic review and meta-analysis	2410	Beta-blockers reduced mortality in comparison to control (RR 0.65, 95%; CI 0.53–0.79; p < .0001)	Beta-blockers reduced mortality without affecting MAP or vasopressor requirement.
Zhang et al. (21)	Meta-analysis	503	Esmolol decreased 28-day mortality; no significant effect in LOS; MAP, CVP, ScvO2 or lactic acid.	Esmolol reduced cardiac troponin I and CO, with no impact on SV, MAP, serum lactate, or ScvO2.
Tan et al. (22)	Systematic review	56,414	Two retrospective studies showed a reduction in mortality with β-blocker exposure. One study demonstrated that β-blockade during sepsis is associated with reduction in mortality.	Beta-blockers reduced the mortality when used prior to or during the sepsis.
Lee et al. (23)	Systematic review	1,030	All trials displayed beneficial effects on HR without any impact on BP; of the six trials that assessed mortality, four showed a reduction.	Beta-blockers improved survival, increased VFD, and decreased ICU LOS
Li et al. (24)	systematic review and meta-analysis	363	Heart rate in β-blocker was significantly less than that in the control group (SMD = −2.01, 95% CI: −3.03, −0.98; P = 0001, I2 = 93%) For MAP and CVP, no significant difference was reported between β-blocker and control	Beta-blockers were proven to reduce mortality, and stabilize the heart rate, with no consequence on tissue perfusion.
Rothenberg et al. (25)	Systematic review	Studies that discussed BB in sepsis: 154 40 8,087	Esmolol showed a reduction in HR by 18 bpm, maintained for 96 hours, and a reduction of 28-day mortality rate	Esmolol showed better control of heart rate and reduction in mortality rate.
Chacko et al. (10)	Systematic review	1,334	6 studies showed improvement in HR control with beta- blockers.	Beta-blockers reduced the HR.
Sanfilippo et al. (26)	Systematic review	343	All studies showed a decrease in HR with beta-blockers; one study showed better hemodynamic parameters with the combination of metoprolol and milrinone.	Esmolol decreased HR in septic patients and in combination with milrinone improved hemodynamics.

**Table 2:** Included systematic reviews and meta-analysis studies regarding aspirin and heparin.

**References**	**Study Design**	**Number of participant s [n]**	**Results**	**Outcomes**
Li et al. (27)	Meta-analysis	684	LMWH reduces the PT [mean difference (MD) =-0.48, 95% when compared to the control group	LMWH reduces the 28-day mortality [relative risk (RR) =0.52] and also decreases the levels of TNF-alpha, D-dimer, and the incidence of MODS.
Ouyang et al. (28)	Meta-Analysis	689,897	Studies used aspirin for antiplatelet therapy, and subgroup analysis showed reduced ICU or hospital mortality in patients with sepsis (OR = 0.60, 95% CI: 0.53–0.68, p < 0.05). Antiplatelet administration drugs can reduce mortality when administered either before (OR = 0.78, 95% CI: 0.77– 0.80) or after sepsis (OR = 0.59, 95% CI: 0.52–0.67).	The use of antiplatelet can reduce the mortality of patients with sepsis, but when aspirin is excluded the effect of clopidogrel was not significant except in one study Subgroup analysis showed that the antiplatelet drugs can reduce mortality both when administered either before or after sepsis begins.
Fan et al. (29)	Meta-analysis	594	The use of LMWH can determine the PT, platelet, d-dimer, pro- inflammatory cytokines interleukin-6 (IL-6), tumor necrosis factor-α (TNF-α) after 7 and 14 days.	LMWH when compared with the usual treatment reduces the 28-day mortality (RR 0.72;) whit no difference in the ICU LOS (MD −2.26 days;). Also LMWH reduces the serum IL-6 levels (SMD −1.76), the levels of TNF-alpha (MD −16.72). LMWH significantly increased platelet counts (MD 18.33; 95%), reduces the PT (MD −0.88; 95%) and there was no difference in the D-dimer levels in the groups studied.
Zarychanski et al. (12)	Systematic review	2,637	The absolute risk for mortality was reduced by 0.88% in placebo and 3.48% in the heparin group.	The use of heparin in critically ill patients with sepsis or septic shock is safe and decreases mortality. More studies related to its safety need to be conducted.
Du et al. (30)	Meta-analysis	5166	In septic patients, antiplatelet therapy was associated with a decreased risk of ICU and hospital mortality,	Antiplatelet therapy was associated with a decreased risk for sepsis, ICU, and hospital mortality. The 28- and 30-day mortalities were not affected by antiplatelet therapy
Trauer et al. (31)	Meta-analysis	Cohorts (n = 17,065), Insurance-based database (n = 683,421).	Hospital-based studies using propensity scores revealed an overall effect of a 6% reduction in mortality. Re-analyzed insurance database study pooled with the hospital-based studies, showed a reduction in mortality of 6%, (95%CI, 2 to 10%; p=0.004)	Antiplatelet drugs and aspirin usage prior to the onset of sepsis are associated with a reduction in mortality.
Rothenberg et al. (25)	Systematic review	Studies that discussed aspirin:	Aspirin users had a significantly reduced mortality compared to non-user.	Aspirin appears to reduce acute lung injury, occurrence of ARDS, and may also impact mortality in critically ill patients. The most severely ill may obtain the most benefit.
		7,945		
		1,149 979		Aspirin significantly reduces mortality, with benefit in the sepsis-only group. This supports the proposal that selected critically ill patients with a high severity of illness score may benefit the most from aspirin use

**Table 3:** Included systematic reviews and meta-analysis studies regarding statins.

**References**	**Study Design**	**Number of participants [n]**	**Results**	**Outcomes**
Pertzov et al. (18)	Systematic review and meta-analysis	2,628	Statins did not reduce 30-day all-cause mortality neither in all patients (RR) 0.96, 95% confidence interval (CI) (0.83– 1.10), nor in a subgroup of patients with sepsis (RR 0.97, 95% CI 0.84– 1.12).	No evidence supports the use of statins in septic patients.
Feng et al. (32)	Systematic review and meta-analysis	9,309	The effect of statins treatment on SOFA score associated with significant reduction score (p < 0.00001).	The sample size was too small to accurately assess the effects on mortality.
Chen et al. (33)	Meta-analysis	2,333	There was no statistically significant difference (RR, 0.90; 95% CI, 0.73–1.11).	Statins therapy did not reduce mortality in septic patients.
Han et al. (34)	Meta-analysis	915	Combination of ulinastatin and Tα1 reduced the all-cause mortality in 28-day (RR 0.67; 95% CI 0.57 to 0.80) and 90-day (RR 0.75; 95% CI 0.61 to 0.93), TNF-α (WMD - 73.86 ng/L; 95% CI - 91.00 to−56.73 ng/L), IL-6 (WMD - 55.04 ng/L; 95% CI - 61.22 to−48.85 ng/L), length of MV (WMD - 2.26 days; 95% CI - 2.79 to−1.73 days) in septic patients compared to placebo.	Immunomodulatory therapy combining ulinastatin and thymosin alpha 1 (Tα1) significantly improves all-cause mortality, inflammatory mediators, and the time needed for mechanical ventilation in septic patients
Tralhão et al. (35)	Systematic review	96,750	In 16 studies statins improved morbidity or reduced mortality (adjusted OR, 0.06– 0.62). In 12 studies and 5 RCT there was no protective effect associated with statins use.	Data do not support statins use in septic patients.
Wan et al. (36)	Meta-analysis	338,515	Among RCT statins did not decrease hospital mortality (RR, 0.98; 95% (CI), 0.73 to 1.33) or 28-day mortality (RR, 0.93; 95% CI, 0.46 to 1.89). Observational studies indicated that statins were associated with a decrease in mortality with adjusted data (RR, 0.65; 95% CI, 0.57 to 0.75) or unadjusted data (RR, 0.74; 95% CI, 0.59 to 0.94).	Limited evidence supporting the significant reduction in mortality
Rothenberg et al. (25)	Systematic Review and meta-analyses	18,102	No RCT showed mortality reduction, except in patients who were prior users of statins (OR 0.17 (0.03 to 0.85), p=0.03).	The use of statins does not reduce mortality in septic patients.
Thomas et al. (37)	Systematic review and meta-analysis	1,818	Overall analysis including 1,818 patients total from four studies showed that there was no difference in 60-day mortality between statins (223/903) and placebo (233/899) [risk ratio, 0.930; 95% CI, 0.722 to 1.198] and no difference in 28-day mortality was observed between groups (statins 191/907, placebo 199/911; risk ratio 0.953; 95% CI, 0.715 to 1.271).	Usage of statins therapy should not be recommended in the management of severe sepsis in critically ill patients for many reasons. There was no association of statins continuation with organ failure-free days and cessation of statins may cause an inflammatory rebound leading to worse outcomes due to their influence on plasma cortisol levels.

HR, heart rate; CO, cardiac output; CVP, central venous pressure; DBP, diastolic blood pressure; CO, cardiac output; ICU, intensive care unit; LOS, length of stay; MAP, mean arterial pressure; MV, mechanical ventilation; RCT, randomized controlled trial; SV, stroke volume; SS, septic shock; SBP, systolic blood pressure; VFD, ventilator-free days.

## Discussion

Management of sepsis is composed of fluid resuscitation, antimicrobial agents, source control, and organ support. However, recently the possibility of adjunctive treatments came into account depending on the pathophysiology of this disease (5). This study aimed to discuss four drugs basically related to heart disease management, but their mechanism of action brought them into consideration in the literature regarding sepsis management. We will discuss each drug by the use rationale and summarize the latest studies that investigated its use in sepsis.

### Beta-blockers

Sinus tachycardia and atrial fibrillation are independent risk factors for mortality in septic patients (20, 38). Septic patients have high sympathetic stimulation due to increased endogenous and exogenous catecholamine concentrations (20). Exogenous catecholamines such as norepinephrine and dobutamine may increase the risk of tachyarrhythmia and thus mortality (39, 40). Decatecholaminisation by beta-blockers inhibits cardiogenic derangements (22), and controlling HR to be less than 95 beats per minute within 24 h improves outcomes (20), while beta-1 receptor blockade reduces HR. Moreover, the chronic use of beta-blockers before sepsis can improve survival (10, 22, 41). Similarly, many studies showed a reduction in mortality when beta-blockers were used during sepsis (20–25, 38, 41). Although a prospective cohort study that recruited 190 patients showed a non-significant reduction in 28-day mortality in the group of esmolol in comparison to the control group, which could be due to the small sample size, there was a significant control of HR, reduction in mechanical ventilation use, and improvement on tissue perfusion (42). Both selective and non-selective beta-blockers were reported to reduce mortality, and the use of ultrashort-acting cardio-selective beta-1 blockers could be important because of their rapid onset and short half-life (20, 25).

Sepsis-induced tachycardia contributes to cardiovascular failure, myocyte death, and dangerous arrhythmias; in this context, beta-blockers can reduce markers of cardiac injury (10) and CO, with no impact on stroke volume (10, 21). The reduction of CO could be due to a better control of HR, and the improvement in SV could be due to increased end-diastolic volume and improvement in contractility, which are also related to a controlled HR (10). Despite these findings, the effects of beta-blockers on cardiac biomarkers are heterogeneous (20). Other benefits attributable to beta-blockers use in sepsis are an increase in VFD (20, 23, 42) and a decrease in ICU LOS (21, 23, 25).

Regarding the concern of the effect of beta-blockers on tissue perfusion and oxygen utilization, two meta-analyses showed that these drugs did not impact lactate levels and ScvO2 (21, 24).

A meta-analysis that included seven RCTs using ultrashort-acting selective beta-blockers in sepsis showed a decrease in serum lactate levels in the esmolol and landiolol groups in comparison to the controls (38). These benefits in tissue perfusion were confirmed by studies with esmolol, showing a decrease in serum lactate and the central venous-to-arterial carbon dioxide difference (PcvaCO2 gap) (30, 42).

Despite beta-blockers did not cause a significant difference in mean arterial pressure in various reports, (20, 21, 30, 38, 42–44), Heliste et al. reported hypotension as an adverse event following beta-blocker administration, and they indicated proper monitoring of BP with their use (20).

An interesting finding is that beta-blockers did not increase the need for vasopressors (20, 21, 38), and surprisingly, an RCT showed a decrease in the vasopressor requirements (45).

### Aspirin and heparin

Sepsis is an acute hypercoagulable state associated with platelet consumption and massive thrombosis leading to venous thromboembolism and disseminated intravascular coagulopathy (DIC) (31).

Aspirin is widely used to prevent and treat vascular diseases such as myocardial infarction and stroke, by irreversibly inhibiting cyclooxygenase-1 which is a key component in the activation of platelets aggregation through thromboxane A2 production (11).

A study examined a low-dose aspirin strategy as a method to limit organ failure by interrupting the coagulation cascade. Aspirin reduced mortality in critically ill patients and this could be related to its role in coagulopathy treatment (25).

Similarly, Ouyang et al. and Rothenberg et al. reviewed the effect of antiplatelet use on the prognosis of patients with sepsis and showed that the use of these drugs can reduce mortality in patients with sepsis (25, 28, 46). This supports that selected critically ill patients with a high illness severity may benefit from aspirin use (25). Consistently, the potential benefits of aspirin use in septic patients were further supported by Du et al., who demonstrated that the antiplatelet therapy was associated with a decreased risk of hospital and intensive care unit (ICU) mortality, but 28- and 30-day mortality were not affected by antiplatelet therapy (47). Moreover, a meta-analysis that included 15 studies that described hospital-based cohorts and one that was a large insurance-based database found a reduction in mortality ranging from 2% to 12% among patients hospitalized for sepsis who had prior aspirin treatment. In addition, a combined analysis of all available observational data showed that antiplatelet drugs and aspirin usage in isolation prior to the onset of sepsis are associated with a reduction in mortality. However, we should account that the large sample size in the insurance database study had a considerable impact on this conclusion (31).

Regarding the timing of aspirine usage in sepsis, a subgroup analysis showed that the antiplatelet drugs can reduce mortality both when administered either before “preventive” (OR = 0.78) or after sepsis “additional” (OR = 0.59) (28). A 10-year retrospective population-based cohort study involving approximately 53,000 sepsis hospital admissions revealed that current aspirin users, who had taken aspirin within 90 days prior to admission, had a lower 90-day mortality rate and a longer mean survival time than past users and non-users (48).

On the other hand, it was found that aspirin therapy was associated with negative secondary outcomes such as a higher risk of ICU-acquired sepsis, increased mechanical ventilation duration, ICU LOS, and thus increased morbidity (31). Ouyang et al. found that the secondary outcomes, which included the duration of mechanical ventilation, the incidence of acute respiratory distress syndrome (ARDS), and the incidence of AKI, needed for renal replacement therapy (RRT) and ICU LOS varied and most of them showed no significant difference (28). Nevertheless, other researchers found that aspirin appears to reduce acute lung injury, occurrence of ARDS, and mortality in critically ill patients and that the most severely ill patients may obtain the most benefit (25); however, these results need to be confirmed by further clinical trials.

Heparin is an anticoagulant drug used to prevent venous thromboembolism (VTE) events by augmenting the effect of antithrombin III, which inhibits several clotting factors, mainly the level of Xa and IIa (1:1 ratio) factors (49).

Comparing heparin to placebo, a systematic review concluded that the risk ratio for mortality in septic patients treated with heparin was reduced in comparison to the control group (12). Consistently, some studies demonstrated that the use of heparin decreases the 28-day mortality [odds ratio (OR) = 0.59] and the multi-organ dysfunction syndrome MODS (OR = 0.32,) in septic patients compared to the control group (27, 29). In addition, a clinical trial conducted on an extracorporeal blood filter (Seraph 100) containing heparin resulted in a faster resolution of bloodstream infections when added to antibiotic therapy for patients with bacteremia (50).

However, the positive effect of heparin use in sepsis is debated. Although some authors reported shorter ICU stays of septic patients treated with heparin, others found no significant difference (29). Despite the positive outcomes of heparin use in septic patients' mortality, it increases the incidence of bleeding when compared to the usual method of treatment (27, 29). Moreover, a meta-analysis conducted on 1,340 patients showed no significant differences in mortality and bleeding complications in septic DIC patients treated with anticoagulants, one of which is heparin, compared to placebo (51).

Sepsis-associated DIC is considered a main indication for anticoagulant use in septic patients. A study conducted with concomitant usage of heparin and antithrombin (AT) supplementation in 15.7% of 159 septic patients with DIC found that heparins were related to a higher survival rate than those who were treated without heparins, but the difference was not statistically significant (84.0% vs. 70.9%, *P* = 0.22) (52).

Moreover, heparin is associated with a lower rate of mortality in DIC positive with high-risk septic patients (SOFA score 13–17), but not in the low-risk to moderate-risk patients (SOFA score ≤ 12). Furthermore, Kazuma et al. demonstrated better outcomes in septic patients with organ dysfunction than those without organ dysfunction treated with anticoagulant. Although the differences were not statistically significant, bleeding was the major unfavorable outcome associated with anticoagulant administration (53).

### Statins

Statins, also known as HMG-CoA reductase inhibitors, are a commonly prescribed class of drugs to lower cholesterol levels in the blood. Statins reduce the risk of cardiovascular diseases by lowering low-density lipoprotein cholesterol (LDL-c) and increasing high-density lipoprotein-cholesterol (HDL-c). The main mechanism of statins is by competitively inhibiting the active site of the rate-limiting enzyme in the mevalonate pathway, HMG-CoA reductase, thus preventing the conversion of HMG-CoA to mevalonic acid, which reduces cholesterol synthesis within the liver and consequently the levels in the bloodstream. In addition to their cholesterol-lowering effects, statins have been demonstrated to improve endothelial function, stabilize atherosclerotic plaques, and have anti-inflammatory, immunomodulatory, and antithrombotic effects (54).

On one hand, limited studies revealed the benefits of statins use in septic and critically ill patients. An immunomodulatory therapy combining ulinastatin and thymosin alpha 1 (Tα1) significantly improves all-

cause mortality, inflammatory mediators, and the time needed for mechanical ventilation in septic patients (34). A cohort study analyzing data from Taiwan's National Health Insurance Research Database found that simvastatin and atorvastatin were associated with improved 30-day survival in sepsis patients, while rosuvastatin did not show efficacy in preventing mortality. This study suggests that the impact of statins on sepsis outcome is associated with drug-specific effects rather than with statins' lipid-lowering potency (55). A systemic review and meta-analysis suggested a possible benefit of statins use by lowering SOFA among septic patients, as well as an increase in VFD, but mortality and sepsis risk remained the same. Similarly, statins were found to decrease the need for ventilation among ARDS patients. Despite the mentioned results, the sample size was too small to accurately assess the effects on mortality and sepsis (32). Moreover, the pleiotropic benefit effect of statins including antioxidant, anti-inflammatory, and antithrombotic effects may contribute to nephroprotection in AKI in the ICU (56).

On the other hand, several studies showed no effect of statins in reducing mortality when compared to placebo. In one meta-analysis based on nine prospective randomized trials, it was shown that statins therapy did not reduce mortality in septic patients compared with placebo-controls (33). A systemic review concluded that there were insufficient results supporting statins use in septic patients due to inconsistent mortality reduction, which was evident in the studies collected (35). Another systemic review and meta-analysis recommended caution in using statins in treating infections and sepsis as there seems to be limited evidence supporting the significant reduction in mortality (36). Consistently, a prospective, randomized, and controlled trial showed that statins appeared not to have any benefit in reducing mortality or illness severity in septic patients. Thus, recent systemic reviews and meta-analyses do not recommend the use of statins in reducing mortality in patients with sepsis. As a result, this impacts patients who have other indications for statins, such as cardiovascular disease (25).

Along with these findings, other studies recommended against the usage of statins in sepsis therapy. A systemic review and meta-analysis study of 1,818 patients confirmed that the usage of statins therapy should not be recommended in the management of sepsis (33). The use of statins therapy in adults for the indication of sepsis is not recommended, as results showed no reduction in 30-day all-cause mortality neither in all patients nor in a subgroup of patients who used statins in sepsis therapy (18).

Regarding the use of statins in septic patients with organ dysfunction, Joannidis et al. concluded that there was no improvement in clinical outcomes or 60 days mortality between septic patients with ARDS treated using rosuvastatin when compared to a placebo (56). Consistently, a prospective study showed an increased mortality rate and no effect of rosuvastatin on the outcomes in patients with sepsis-associated ARDS (18). Moreover, there were no consistent findings in a study assessing statins for ALI in septic patients using rosuvastatin (57). Additionally, there was no association of statins continuation with organ failure-free days (37), and rosuvastatin use showed small differences in organ failure-free days when compared to a placebo suggesting a specious argument in their effect on organ failure prevention (56). Regarding liver enzymes, it was reported that aspartate aminotransferase (AST) levels in patients taking rosuvastatin for sepsis associated with ARDS were elevated. Although they concluded that rosuvastatin was not associated

with the increase in serum creatinine kinase levels, some patients with elevated AST levels were having related elevations in creatine kinase and alanine aminotransferase (ALT) levels, which suggested the contribution of rosuvastatin in renal and hepatic failure (56). Furthermore, statins therapy was shown not to influence the plasma cortisol profiles in patients with severe sepsis leading to prolonged inflammatory response, hospital stay, and morality (58). However, the cessation of statins may cause an inflammatory rebound leading to worse outcomes due to their influence on plasma cortisol levels (37).

## Conclusion

Our narrative review does not recommend either in favor or against using beta-blockers, aspirin, heparin, or statins in the management of sepsis. However, beta-blockers showed benefits in improving hemodynamics and reducing mortality and their safety on tissue perfusion supporting their use in selected septic patients.

The target hemodynamic parameters and the choice of beta-blocker need further studies. Additionally, aspirin and heparin have promising feedback on improving survival, but more studies are needed to support their use. Most studies recommended against the use of statins in septic patients. In conclusion, there were variable results regarding these drug effects on sepsis outcome, mortality, and prognosis. We strongly suggest further studies be conducted on this field to clear the controversy.

## Limitations

This study was limited in several ways: (1) the potential confounding factors such as the varying dose and treatment period of each drug; (2) the combination with other drugs as anticoagulant increase the heterogeneity among the included study; (3) as sepsis is a polygenic disease, we did not approach the individual and genomic influence on pharmacological actions in septic patients; (4) in addition to the language limitations, only databases in English were concluded in this study.

## Author contributions

AA, LA-H, and FD conceived and designed the manuscript. AA, HA, BA, MA, OQ, ZA, GF, SH, and IQ analyzed the data and wrote the manuscript. AA, HA, LA-H, FD, and GC reviewed and edited the data analysis and writing. All authors have read and approved the final version of this manuscript.
